# Targeting Cancer Stem Cells with Differentiation Agents as an Alternative to Genotoxic Chemotherapy for the Treatment of Malignant Testicular Germ Cell Tumors

**DOI:** 10.3390/cancers13092045

**Published:** 2021-04-23

**Authors:** Amanda R. Loehr, Timothy M. Pierpont, Eric Gelsleichter, Anabella Maria D. Galang, Irma R. Fernandez, Elizabeth S. Moore, Matthew Z. Guo, Andrew D. Miller, Robert S. Weiss

**Affiliations:** 1 Department of Biomedical Sciences, Cornell University, Ithaca, NY 14853, USA; arl244@cornell.edu (A.R.L.); eg338@cornell.edu (E.G.); adg88@cornell.edu (A.M.D.G.); irf25@cornell.edu (I.R.F.); mg943@cornell.edu (M.Z.G.); andrew.miller@cornell.edu (A.D.M.); 2 Department of Microbiology and Immunology, Cornell University, Ithaca, NY 14853, USA; tmp68@cornell.edu; 3 Department of Biomedical Engineering, Cornell University, Ithaca, NY 14853, USA; esm84@cornell.edu

**Keywords:** testicular germ cell tumor, embryonal carcinoma, nonseminoma, differentiation therapy, thioridazine, cancer stem cells, pluripotency

## Abstract

**Simple Summary:**

Testicular germ cell tumors (TGCTs) are the most common solid malignancy in young men. Fortunately, these tumors are highly curable with conventional genotoxic chemotherapy. However, because these chemotherapeutics have significant short- and long-term side effects, less toxic treatment options are desired. We report that thioridazine, an FDA-approved antipsychotic, differentiates embryonal carcinoma (EC) cells, the cancer stem cells of many malignant TGCTs, and suppresses their tumor propagating activity. Additionally, thioridazine treatment of mice with EC-containing neoplasms extends survival relative to untreated control mice. These findings identify thioridazine as a promising alternative or adjuvant to chemotherapy for the treatment of TGCTs.

**Abstract:**

Testicular germ cell tumors (TGCTs) are exceptionally sensitive to genotoxic chemotherapy, resulting in a high cure rate for the young men presenting with these malignancies. However, this treatment is associated with significant toxicity, and a subset of malignant TGCTs demonstrate chemoresistance. Mixed nonseminomas often contain pluripotent embryonal carcinoma (EC) cells, the cancer stem cells (CSCs) of these tumors. We hypothesized that differentiation therapy, a treatment strategy which aims to induce differentiation of tumor-propagating CSCs to slow tumor growth, could effectively treat mixed nonseminomas without significant toxicity. The FDA-approved antipsychotic thioridazine and the agricultural antibiotic salinomycin are two drugs previously found to selectively target CSCs, and here we report that these agents differentiate EC cells in vitro and greatly reduce their tumorigenic potential in vivo. Using a novel transformed induced pluripotent stem cell allograft model and a human xenograft model, we show that thioridazine extends the survival of tumor-bearing mice and can reduce the number of pluripotent EC cells within tumors. These results suggest that thioridazine could be repurposed as an alternative TGCT treatment that avoids the toxicity of conventional chemotherapeutics.

## 1. Introduction

Testicular germ cell tumors (TGCTs) comprise nearly 95% of all testicular cancers [1]. The most common TGCTs arise from a precursor lesion known as germ cell neoplasia in situ (GCNIS) [2]. GCNIS-related TGCTs are described as either seminomas (~40% of TGCTs) or nonseminomas (~60%), with this distinction being critical for making treatment decisions [2,3]. Nonseminomas are often mixed tumors, containing some combination of choriocarcinoma, yolk sac tumor, embryonal carcinoma, or teratoma components, and they are often more aggressively metastatic than seminomas [1,4,5].

Fortunately, the majority of TGCTs exhibit an exceptional sensitivity to conventional genotoxic chemotherapy, contributing to an overall five-year survival rate of nearly 95% [6,7,8]. The exact mechanism of their chemosensitivity remains unresolved, but it is likely multifactorial and coupled to the germ cell ontogeny of TGCTs [9,10]. Although contemporary treatment regimens yield a high cure rate, some TGCT patients nonetheless succumb to cisplatin-resistant or relapsing metastatic disease. Metastatic TGCTs are identified in 30% of patients at the time of their initial diagnosis [2]. Between 15% and 20% of these patients will relapse following first-line chemotherapy, only half of which can be rescued by salvage efforts [11].

Chemotherapy is often temporarily noxious and sometimes permanently debilitating. Acute side-effects such as nausea, vomiting, diarrhea, constipation, oral and gastrointestinal mucositis, and hypersensitivity reactions can be pharmacologically mitigated but are unavoidable to some degree [12,13]. As TGCT patients are relatively young and often cured, TGCT survivors face significant long-term morbidity associated with chemotherapy, such as neurotoxicity, nephropathy, cardiovascular disease, pulmonary damage, secondary malignancies, metabolic syndrome, hypogonadism, psychosocial difficulties, and subfertility [14,15,16]. Furthermore, compared to TGCT patients who receive surgery alone, those given cisplatin-based chemotherapy have an increased risk of hearing impairment, tinnitus, peripheral neuropathy, and Raynaud’s syndrome [17]. Accordingly, less toxic and more efficacious alternatives to chemotherapy are needed.

Differentiation therapy is a potential alternative to chemotherapy, seeking to exploit a non-cytotoxic developmental mechanism to prevent cancer progression. The cancer stem cell (CSC) concept specifies that CSCs are responsible for tumor growth, invasion, and dissemination [18,19,20,21]. Differentiation therapy seeks to specifically induce the differentiation of CSCs, thereby eliminating or attenuating their potential for proliferation and metastasis [18]. Mixed nonseminomatous TGCTs are often composed of pluripotent embryonal carcinoma (EC) cells and differentiated teratoma cells [22]. EC cells are the CSCs of these tumors, and although they are generally responsible for the unique chemosensitivity of TGCTs [23], they can display or develop chemoresistance and thus contribute to the development of residual tumors or disease relapse [3,24]. Consequently, selectively inducing EC cell differentiation might offer an attractive alternative or adjunctive means of treating TGCTs, especially those that are multirelapsing or otherwise chemoresistant.

Efforts to develop CSC-targeted therapies have included several large drug screens. One such study by Gupta et al. found that salinomycin, an antibiotic used in agriculture [25], displays selective toxicity for breast CSCs and inhibits mammary tumor growth in vivo [26]. A few years later, thioridazine, an FDA-approved antipsychotic, was shown to: (1) selectively induce the differentiation of neoplastic human pluripotent stem cells (hPSCs) while sparing nonneoplastic hPSCs and (2) selectively induce the differentiation and reduce the in vivo function of the CSCs that cause acute myeloid leukemia (AML) without affecting normal hematopoietic stem cell function [27]. Notably, schizophrenia patients treated with dopamine-receptor antagonists experience a reduced incidence of colorectal and prostate cancer, and dopaminergic-deficient Parkinsonian patients have a decreased risk for a variety of cancers [28,29]. Therefore, thioridazine is an accessible and well-characterized drug that might readily translate into an alternative or adjunctive TGCT therapeutic.

Given the exceptional success of treating TGCTs with genotoxic drugs, the clinical exploration of alternative therapies must be justified by evidence from robust and predictive preclinical animal models. Our lab developed a genetic mouse model of spontaneous, in situ, malignant TGCTs, which we have termed gPAK mice (i.e., germ cell-specific *Pten* and *Kras* mutant mice) [30]. Following in utero germ cell-specific malignant transformation, these mice develop malignant, often metastatic, teratocarcinomas with histopathological and molecular features reminiscent of the mixed nonseminomas typically encountered in young-adult human patients [23]. Importantly, similar to TGCTs in humans, the autochthonous TGCTs of gPAK mice originate during a restricted period of embryonic development and are highly responsive to standard chemotherapeutic regimens [23]. These attributes make the gPAK model an invaluable tool for studying human TGCT pathogenesis and for evaluating novel therapeutic strategies and diagnostic frameworks for TGCTs.

We sought to investigate the therapeutic potential of differentiation therapy using the gPAK model, as well as murine allograft and human xenograft models. We hypothesized that thioridazine and salinomycin would induce differentiation of the murine EC cells and that the antineoplastic effects of thioridazine would improve the survival of mice bearing malignant teratocarcinomas. Herein, we report that: (1) thioridazine and salinomycin differentiate EC cells and reduce their tumorigenic potential, and (2) thioridazine treatment of tumor-bearing mice extends survival and reduces the number of pluripotent CSCs within tumors. These data suggest that differentiation therapy is a promising treatment strategy for mixed nonseminomas that should be further explored.

## 2. Materials and Methods

### 2.1. Mouse Strains and Husbandry

All animals used in this study were handled in accordance with federal and institutional guidelines, under a protocol approved by the Cornell University Institutional Animal Care and Use Committee. Mice were housed in an Association for the Assessment and Accreditation of Laboratory Animal Care International-accredited facility and cared for in compliance with the Guide for the Care and Use of Laboratory Animals [31]. gPAK mice were generated as previously described [23]. The breeding strategy used generates tumor-prone mice that either lack *Pten* alone or have both *Pten* deletion and *Kras* activation in germ cells. Nude (NU/J, #002019) and Rag2KO (B6(Cg)-Rag2^tm1.1Cgn^/J, #008449) mice were obtained from The Jackson Laboratory and bred in our facilities. Mice were monitored regularly for health status and were euthanized by CO_2_ asphyxiation upon reaching humane endpoint criteria (tumor greater than 2 cm in diameter, tumor preventing ambulation, ulceration of skin around tumor, loss of over 20% of body weight, dehydration that does not improve with dietary supplementation, and/or signs of chronic pain such as a hunched posture, piloerection, and poor grooming).

### 2.2. Genotyping

Genotyping was performed by polymerase chain reaction (PCR) amplification using allele-specific primers and genomic DNA extracted from mouse tails or mouse embryonic fibroblasts (MEFs). *Pten* and *Kras* alleles and *Stra8-Cre* and *OCT4-gfp* transgenes were determined as previously described [30]. Identification of the *Sry* gene was used to determine the sex of MEFs (F 5′-CCA CTC CTC TGT GAC ACT TTA GCC CGA-3′, R 5′-TTG TCT AGA GAG CAT GGA GGG CCA TGT CAA-3′).

### 2.3. Primary EC Cell Culture and Differentiation

TGCTs from gPAK mice [23] were isolated and disaggregated with 2 mg/mL Collagenase A (Roche, Basel, Switzerland) in serum-free DMEM (Corning Inc., Corning, NY, USA) with penicillin, streptomycin, and gentamycin at 37 °C for approximately 1 h with shaking. Cells were then passed through a 40 µm filter to remove large clumps, pelleted at 1.5× *g* for 5 min, resuspended in DMEM containing 10% FBS (Sigma, St. Louis, MO, USA) with 1× LIF (ORF Genetics, Kopavogur, Iceland) and 10 μM ROCKi (Selleckchem, Houston, TX, USA) and plated at various densities into 10-cm tissue culture plates. Cultures were passed at confluence until EC colonies were visually observed, followed by subsequent passages to propagate each cell line. EC3 was derived from a *Pten^−/−^ Kras^+/+^ Stra8-Cre*^Tg^ *OCT4-gfp*^Tg^ tumor, EC11 from a *Pten^−/−^ Kras^+/+^ Stra8-Cre*^Tg^ tumor, and EC14 from a *Pten^−/−^ Kras^+/^*^G12D^ *Stra8-Cre*^Tg^ *OCT4-gfp*^Tg^ tumor. EC cell lines were maintained in EC medium (DMEM + 15% heat-inactivated Bovine Growth Serum (Hyclone, Logan, UT, USA) + 2% L-glutamine (Corning) + 1% nonessential amino acids (Corning) + 1% Pen/Strep (Corning) + β-mercaptoethanol + 1 × LIF). Media were replaced daily, and cells were passaged by trypsinization 1:10 every other day. To induce differentiation, cells were incubated with thioridazine hydrochloride (Sigma) (10 μM for TR14 and TR3, 20 μM for TR11) or salinomycin (sodium salt) (Cayman Chemical Company, Ann Arbor, MI, USA) (2 μM for SAL14, 4 μM for SAL11), in media without LIF, for up to 2 weeks or until EC colonies were no longer observed, at which point the cultures were maintained in EC media without LIF.

### 2.4. Culture of Human EC Cell Lines

Human EC cell lines NTERA-2 cl.D1 (NT2D1) and NCCIT were obtained from ATCC (Manassas, VA, USA). NT2D1 cells were maintained in DMEM + 15% heat-inactivated Bovine Growth Serum + 2% L-glutamine + 1% nonessential amino acids + 1% Pen/Strep and were passaged 1:4 when confluent by scraping. NCCIT cells were maintained in RPMI-1640 + 15% heat-inactivated Bovine Growth Serum + 2% L-glutamine + 1% nonessential amino acids + 1% Pen/Strep and were trypsinized and passaged 1:8 when confluent. NT2D1 cells were differentiated by culturing with 5 mM hexamethylene bisacetamide (HMBA; Abcam, Cambridge, MA, USA) for up to one week.

### 2.5. Derivation of Transformed iPS Cells

Mouse embryonic fibroblasts (MEFs) were prepared from embryonic day-13.5 embryos from timed matings between *Pten^+/-^* and *Pten*^fl/fl^ *Kras^+/^*^LSL^ mice. Embryos were dissected from the decidua of a pregnant mouse, gently homogenized, and cultured in DMEM + 15% heat-inactivated Bovine Growth Serum + 2% L-glutamine + 1% nonessential amino acids + 1% Pen/Strep. Primary MEFs were passaged according to the 3T3 protocol [32]. For reprogramming, 50,000 early passage male *Pten*^fl/-^ *Kras^+/^*^LSL^ MEFs were seeded in a 6-well plate. The next day, media were replaced with fresh media and viral supernatant (doxycycline-inducible lentivirus expressing *Oct4*, *Sox2*, *Klf4*, and *c-Myc*, prepared as previously described [33]). Two days later, the cells were passed onto a 10 cm dish with EC media + LIF + 2 μg/mL doxycycline hyclate (Sigma). Media were replaced daily. Individual iPS cell colonies were isolated, dissociated with trypsin, plated on feeder cells, and subsequently expanded. Feeder cells were prepared by irradiating wild-type primary MEFs with 35 Gy. An iPS cell line with good colony morphology was made doxycycline-independent and was used for further experiments. For malignant transformation, 1 million iPS cells were plated on feeder cells in a 10 cm dish. The next day, cells were transduced with a Cre-recombinase expressing adenovirus (prepared as previously described [34]) in 2.5 mL culture medium. The next day, media were replaced with fresh EC media + LIF. Both normal and transformed iPS cells were passaged 1:10 every other day and media were replaced daily.

### 2.6. Western Blot Analysis 

Cell lysates were prepared in cell lysis buffer (150 mM NaCl, 50 mM Tris-HCl, pH 8.0, 1 mM EDTA, 1% Nonidet P-40, 0.1% SDS, and 0.5% sodium deoxycholate) supplemented with aprotinin, leupeptin, sodium orthovanadate, and phenyl-methylsulfonyl fluoride. Whole testis lysate was prepared in tissue lysis buffer (140 mM NaCl, 10 mM Tris-HCl, pH 8.0, 1 mM EDTA, 0.5 mM EGTA, 1% Triton X-100, 0.1% SDS, and 0.1% sodium deoxycholate) supplemented with aprotinin, leupeptin, sodium orthovanadate, and phenyl-methylsulfonyl fluoride. Protein concentration was determined by Bradford assay (Bio-Rad, Hercules, CA, USA). Proteins were resolved by SDS-PAGE, transferred to PVDF membranes, and blocked with 5% BSA. Membranes were incubated with rabbit primary antibody (OCT4 [ab18976 1:1000, Abcam]; DRD2 [55084-1-AP 1:1000, Proteintech, Rosemont, IL, USA]; GAPDH [14C10 1:5000, Cell Signaling Technology, Danvers, MA, USA]) overnight at 4 °C, followed by incubation with HRP-conjugated goat anti-rabbit secondary antibody (4030-05 1:5000, SouthernBiotech, Birmingham, AL, USA) for 1 h at room temperature. Bands were visualized on a Bio-Rad ChemiDoc XRS after brief incubation with WesternBright ECL HRP substrate (Advansta, San Jose, CA, USA). ImageJ was used for densitometry analysis.

### 2.7. Quantitative PCR 

RNA was extracted from cells using RNA Stat-60 (Tel-Test, Inc., Friendswood, TX, USA) according to the manufacturer’s protocol. Complementary DNA was synthesized from total RNA using a high-capacity cDNA reverse transcription kit (Applied Biosystems, Waltham, MA, USA). Quantitative PCR was performed using C1000 Touch Thermal Cycler, CFX96 Real-Time System (Bio-Rad). Gene expression levels were normalized to that of the housekeeping gene *Ppia. Pou5f1* (*Oct4*) primers were 5′-GGA AGC CGA CAA CAA TGA-3′ and 5′-CAC GGT TCT CAA TGC TAG TT-3′, *L1td1* primers were 5′-AAG GCG CAG ATT TGG GAT TTT G-3′ and 5′-CCC GAC ATG GTG GAT ACA CTG-3′, and *Ppia* primers were 5′-ACT GAA TGG CTG GAT GGC AA-3′ and 5′-CAA AAC GCT CCA TGG CTT CC-3′. Three independent experiments were performed, each with three technical replicates per cell line.

### 2.8. RNA Sequencing 

RNA was extracted from cells from three independent culture dishes per cell line using RNA Stat-60 according to the manufacturer’s protocol, with the addition of GlycoBlue Coprecipitant (Invitrogen, Waltham, MA, USA) immediately prior to isopropanol precipitation. RNA sample quality was determined by spectrophotometry (Nanodrop) to determine concentration and chemical purity and with a Fragment Analyzer (Advanced Analytical) to determine RNA integrity. Library preparation, sequencing, and analysis was performed by the Transcriptional Regulation and Expression Facility at Cornell University. PolyA+ RNA was isolated with the NEBNext Poly(A) mRNA Magnetic Isolation Module (New England Biolabs, Ipswich, MA, USA). TruSeq-barcoded RNASeq libraries were generated with the NEBNext Ultra II Directional RNA Library Prep Kit (New England Biolabs), quantified with a Qubit 2.0 (dsDNA HS kit, ThermoFisher, Waltham, MA, USA), and the size distribution was determined with a Fragment Analyzer. Libraries were sequenced on a NextSeq500 instrument (Illumina, San Diego, CA, USA). At least 20M single-end 75bp reads were generated per library. Reads were trimmed for low quality and adaptor sequences with TrimGalore v0.6.0, a wrapper for cutadapt [35], and fastQC. Reads were mapped to the reference transcriptome Ensembl GRCm38 using STAR v2.7.0e [36]. SARTools [37] and DESeq2 v1.26.0 [38] were used to generate normalized counts and statistical analysis of differential gene expression. iDEP.91 [39] was used to generate a principal component analysis (PCA) plot and heatmap from normalized counts data, using default settings.

### 2.9. Gene Set Enrichment Analysis

DESeq2 normalized count values were averaged over 3 independent replicates per group. Data were filtered to include only expressed genes, defined as a minimum normalized count of 50 in at least one group. Preranked gene lists were created comparing each EC cell line to its thioridazine-differentiated derivative. To create custom gene sets, The Cancer Genome Atlas data were accessed using cBioPortal [40,41]. Mean log2 expression (as determined by RNASeq) values for 17 pure embryonal carcinomas was compared to those of 3 pure teratomas. We found that 1297 genes were significantly upregulated in the embryonal carcinomas compared to the teratomas (FDR < 0.05) and were included in the custom gene set “TCGA-EC”. We found that 1316 genes were significantly upregulated in the teratomas compared to the embryonal carcinomas (FDR < 0.05) and were included in the custom gene set “TCGA-TERATOMA”. Preranked gene lists and custom gene sets were used for Gene Set Enrichment Analysis (GSEA) [42]. GSEA (preranked) was performed with 1000 permutations and a classic enrichment statistic.

### 2.10. Gene Ontology Analysis

Gene lists containing genes that were significantly upregulated (FDR < 0.05, normalized count ≥ 50 in at least one group, and log_2_(fold change) ≥ 1) in both EC11 and EC14 compared to their differentiated derivatives (804 genes) and those that were significantly upregulated in both TR11 and TR14 compared to their parental EC lines (196 genes) were used in a PANTHER Overrepresentation Test using the GO biological process annotation data set from the GO Ontology database [43]. Significantly overrepresented biological processes were determined by Fisher’s Exact test with Bonferroni correction for multiple testing and were ranked by fold enrichment.

### 2.11. Population Doubling Assay

10 cm plates were seeded with 1 million cells on day zero. Every two days, cells were trypsinized, counted, and reseeded at 1 million cells, for 12 days. Population doubling was calculated using the formula PD = 3.32(logC−logS), where C = the number of cells counted and S = the number of cells initially seeded.

### 2.12. Soft Agar Assay

6-well plates were coated with 0.6% agarose in growth media. Cells were then suspended in 0.3% low melting point agarose in growth media and added to the solidified coated wells (1000 cells per well). Colonies were photographed after 7 days of growth. After 10 days of growth, cells were fixed and stained with 1 mg/mL nitro-blue tetrazolium in an incubator overnight before plate images were taken. Three technical replicates per cell line were performed per experiment. Three independent experiments were performed.

### 2.13. Immunohistochemistry

Tumors were fixed overnight at room temperature in 4% paraformaldehyde (Sigma) in PBS, embedded in paraffin wax, and sectioned at 5 μm. Sections were deparaffinized and rehydrated, and antigens were unmasked via heat-mediated antigen retrieval in EDTA buffer, pH 8.0. Tissue sections were incubated with 1:10 30% H_2_O_2_:methanol to quench endogenous peroxidase, and then blocked with 4% BSA (Sigma) in TBS with 0.02% Tween-20 (Sigma). Sections were incubated in OCT4 primary antibody (Abcam ab19857, 1:1000) overnight at 4 °C in a humidified chamber, followed by biotinylated antirabbit secondary antibody (Histostain SP, Invitrogen) and streptavidin-peroxidase (Vector Laboratories, Burlingame, CA, USA) at room temperature. Immunoreactivity was visualized with DAB (Thermo Scientific, Waltham, MA, USA) and tissues were counterstained with hematoxylin (Fisher CS401-1D) before dehydration and mounting. To count OCT4 positive cells, representative tissue sections stained for OCT4 were digitized using Aperio ScanScope. ImageJ was used to separate positive and negative colors, and code was written to iteratively compile images with cells detected at multiple intensity thresholds and count the cells. Randomized sections were manually checked for accuracy of automated counting. OCT4-positive cell counts were reported as a percentage of all counted cells.

### 2.14. Subcutaneous Cell Transplantations

Cultured cells were trypsinized and counted, and the appropriate number of cells was resuspended in PBS and kept on ice. Mice were anesthetized via isoflurane inhalation. Immediately prior to injection, 100 μL of cell suspension was mixed with 100 μL of cold Matrigel (Corning) and injected subcutaneously into the flanks of mice. Each mouse received bilateral injections. Mice were monitored regularly for overall health and palpated for tumor detection. For tumorgenicity studies in which mice received EC11, TR11, or SAL11 cells, 6–12-week-old male and female Rag2KO mice were each injected bilaterally with 1000 cells, palpated regularly for tumor detection, and collected 3 weeks after the first tumor was detectable at 2 mm in diameter or 60 days post-injection if tumors never formed. For tumorgenicity studies in which mice received EC14, TR14, SAL14, EC3, or TR3 cells, 6-7-week-old male nude mice were each injected bilaterally with 1000 cells, palpated regularly for tumor detection, and collected 2 weeks after the first tumor was detectable at 2 mm in diameter or 60 days post-injection if tumors never formed. For tumorgenicity studies in which mice received normal and transformed iPS cells, 12–16-week-old male nude mice were injected with 1 million normal iPS cells on the left flank, and 1 million transformed iPS cells on the right flank. Mice were collected when a tumor reached 2 cm in diameter. For studies in which mice received transformed iPS cells, 6-16-week-old male and female nude mice were injected bilaterally with 1 million cells. For studies in which mice received NT2D1 cells, 14–20-week-old male and female Rag2KO mice were injected bilaterally with 10 million cells.

### 2.15. Thioridazine Treatment of Tumor-Bearing Mice

Mice that received subcutaneous transplantation of transformed iPS cells or NT2D1 cells were palpated regularly following injection. When a tumor reached approximately 2 mm in diameter, mice were randomly assigned to either the control or thioridazine-treated group. Thioridazine-treated mice received 25 mg/kg of thioridazine hydrochloride (dissolved in sterile 0.9% NaCl at 5 mg/mL and sterile filtered) via intraperitoneal injection every 3 days for 3 weeks (7 doses total) or until humane endpoint criteria were met. Control mice received an equal volume of sterile 0.9% NaCl in the same manner. Mice were monitored regularly for health status and tumor size. Mice that reached endpoint based on the size of the tumor (greater than or equal to 2 cm in any dimension) were included in survival analyses and their tumors were analyzed for OCT4 expression. Mice that reached endpoint based on poor health status that was independent of tumor burden (dehydration, etc.) or that were found dead were included in the survival analysis, but their data were censored at endpoint, and their tumors were not included in histopathologic analyses.

### 2.16. Fertility Study

Wild-type 129S6 mice were randomly assigned to either the control or thioridazine-treated group at weaning. Thioridazine-treated mice received 25 mg/kg of thioridazine hydrochloride (dissolved in sterile 0.9% NaCl at 5 mg/mL and sterile filtered) via intraperitoneal injection every 3 days starting at 21 days of age for 3 weeks (7 doses total). Control mice received an equal volume of sterile 0.9% NaCl in the same manner. At 7 weeks of age, the mice were bred to 2 female littermates each and allowed to mate for 12 weeks.

### 2.17. Statistical Analysis 

When comparing two groups, an F test was performed to compare variances and a Shapiro–Wilk test was performed to assess normality. If variances were not significantly different and the data were determined to be normally distributed, an unpaired, two-tailed *t*-test was performed. If variances were significantly different and the data were normally distributed, a Welch’s *t*-test was performed. If data were not normally distributed, a Mann–Whitney test was performed. When comparing more than two groups, a Brown–Forsythe test was performed to assess equality of variance and a Shapiro–Wilk test was performed to assess normality. If variances were not significantly different and the data were determined to be normally distributed, a one-way ANOVA and Tukey’s test for multiple comparisons was performed. If the data were not normally distributed, a Kruskal–Wallis test and Dunn’s multiple comparisons test were performed. All statistical analyses were performed using GraphPad Prism (San Diego, CA, USA). Specific tests and *p*-values are reported in the figure legends.

## 3. Results

### 3.1. Thioridazine and Salinomycin Induce Differentiation of Murine EC Cells

**Figure 1:**
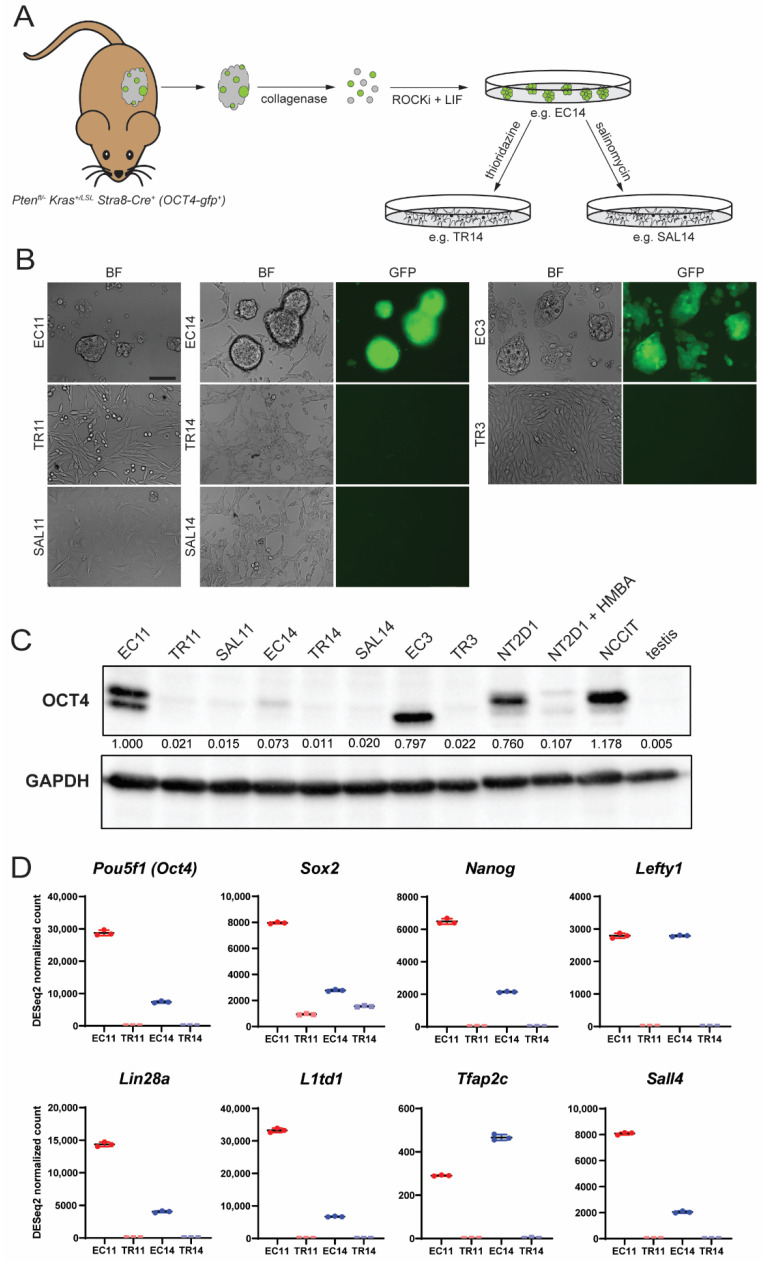
Treatment of murine EC cells with thioridazine or salinomycin alters cell morphology and decreases expression of pluripotency markers. (**A**) Derivation of murine EC cell lines from spontaneous malignant teratocarcinomas in gPAK mice. (**B**) Bright field and fluorescence images of three independent EC cell lines (EC11, EC14, and EC3), two of which carry an OCT4-gfp transgene (EC14 and EC3), before and after thioridazine (TR11, TR14, and TR3)- or salinomycin (SAL11 and SAL14)-mediated differentiation. Scale bar = 100 μm. (**C**) Western blot showing OCT4 protein expression (OCT4A and OCT4B isoforms) in murine EC cell lines, their thioridazine- and salinomycin-differentiated derivatives, human EC cell lines NT2D1 and NCCIT, HMBA-differentiated NT2D1 cells, and murine testis tissue. Densitometry values were normalized to GAPDH (loading control) and expressed as a proportion of the first lane. (**D**) DESeq2 normalized count values from RNASeq data for eight pluripotency-associated genes. Data are mean values ± standard deviation, *n* = 3 independent replicates per group.

In order to test the sensitivity of EC cells to differentiation-inducing agents, we first derived primary EC cell cultures from murine malignant teratocarcinomas [23] (Figure 1A). Tumors were dissociated into single cells and cultured, after which the EC cells formed embryonic stem (ES) cell-like colonies. The EC3 and EC11 cell lines were derived from *Pten-null* tumors, whereas the EC14 cell line was derived from a *Pten-null, Kras*^G12D^ mutant tumor. Additionally, the EC3 and EC14 cell lines contained an *OCT4-gfp* transgene. All three EC cell lines proliferated indefinitely in culture, and those carrying the *OCT4-gfp* transgene were GFP-positive (Figure 1B). When cultured with thioridazine (subsequently named TR3, TR11, and TR14) or salinomycin (SAL11 and SAL14), the cells no longer formed ES-like colonies, appeared more fibroblast-like, and lost *OCT4-gfp* expression (Figure 1B). Similar morphological changes and loss of *OCT4-gfp* expression were consistently observed following multiple independent thioridazine or salinomycin treatments of the EC cell lines. Quantitative PCR (qPCR) analysis revealed a dramatic reduction in the expression of *Pou5f1* (encoding OCT4) and an additional pluripotency marker, *L1td1*, after thioridazine- or salinomycin-mediated differentiation, with thioridazine treatment causing the greater reduction in pluripotency gene expression in most cases (Supplementary Figure S1A). Additionally, Western blot analysis shows that with the exception of EC14, the murine EC cells expressed OCT4 protein at levels similar to the human EC cell lines NT2D1 and NCCIT, and that thioridazine- or salinomycin-differentiated cells displayed a reduction in OCT4 levels to a similar or even greater extent than NT2D1 cells differentiated with HMBA, a known inducer of NT2D1 differentiation [44] (Figure 1C). Interestingly, the murine EC cells differed in the relative abundance of the two OCT4 isoforms detected (Figure 1C). It remains unclear whether there are any functional differences between these and other OCT4 isoforms [45].

To understand the differences between EC cells and their thioridazine-differentiated derivatives at the transcriptome level, we performed RNASeq on two of the three EC cell lines, EC11 and EC14, as well as the matched differentiated counterparts, TR11 and TR14. Principal component analysis and a hierarchical clustering heatmap show that at the transcriptome level, EC11 and EC14 cells were more similar to each other than either one was to its own thioridazine-differentiated derivative (Supplementary Figure S1B,C). Many of the most differentially expressed genes between EC and TR cells were associated with pluripotency, such as *Nanog*, *Lin28a*, and *Sall4* (Figure 1D). Gene ontology (GO) analysis of genes significantly upregulated in both EC cell lines compared to their differentiated derivative revealed overrepresentation of GO Biological Processes such as embryonic placenta development, cellular response to leukemia inhibitory factor, and anterior/posterior pattern specification, emphasizing the pluripotent nature of these cells (Supplementary Figure S2A). GO Biological Processes that were overrepresented in the differentiated cell lines compared to their parental EC cell lines included the regulation of epithelial cell differentiation, circulatory system development, and animal organ morphogenesis, suggesting that these are a more differentiated cell type (Supplementary Figure S2B).

**Figure 2:**
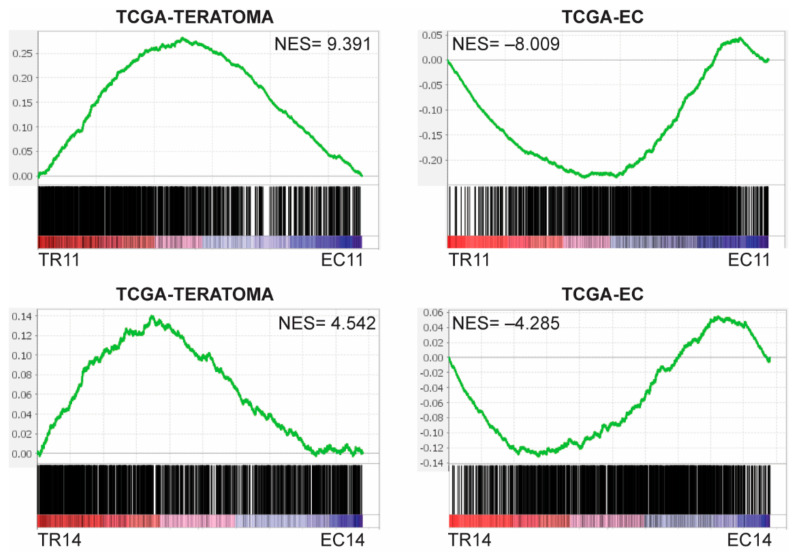
Murine EC cells and their thioridazine-differentiated derivatives transcriptionally resemble human EC and teratoma, respectively. Gene sets were created by comparing RNASeq data from The Cancer Genome Atlas (TCGA) for 17 pure embryonal carcinomas and 3 teratomas. TCGA-EC includes genes significantly upregulated (FDR < 0.05) in the embryonal carcinomas, and TCGA-TERATOMA includes genes significantly upregulated in the teratomas. Preranked gene lists were created comparing each murine EC cell line to its thioridazine-differentiated derivative. Gene Set Enrichment Analysis (GSEA) enrichment plots indicate that genes that are upregulated in human pure embryonal carcinomas (TCGA-EC) were overrepresented in murine EC cells, and genes that are upregulated in human pure teratomas (TCGA-TERATOMA) were overrepresented in the thioridazine-differentiated cells. NES = normalized enrichment score. FDR < 0.001 for all enrichment plots.

We next analyzed how the murine EC cells and differentiated derivatives compared transcriptionally to the EC and teratoma components of human TGCTs, since teratoma is a product of spontaneous EC differentiation in vivo in mixed germ cell tumors. To assess this, we compared the transcriptome data from 13 human embryonal carcinomas to that of 7 pure teratomas in The Cancer Genome Atlas (TCGA), and created gene sets from the genes significantly upregulated in the pure embryonal carcinomas (1297 genes, TCGA-EC) or significantly upregulated in the pure teratomas (1316 genes, TCGA-TERTOMA). Gene Set Enrichment Analysis (GSEA) revealed that EC11 and EC14 cells were enriched for genes in the TCGA-EC gene set, whereas TR11 and TR14 cells were enriched for genes in the TCGA-TERATOMA gene set, all with highly significant normalized enrichment scores (Figure 2). This suggests that on the transcriptome level, the murine EC cells are similar to human embryonal carcinomas, and their thioridazine-differentiated derivatives are similar to human teratomas.

### 3.2. Thioridazine- and Salinomycin-Differentiated Cells Have a Reduced Tumorigenic Potential

**Figure 3:**
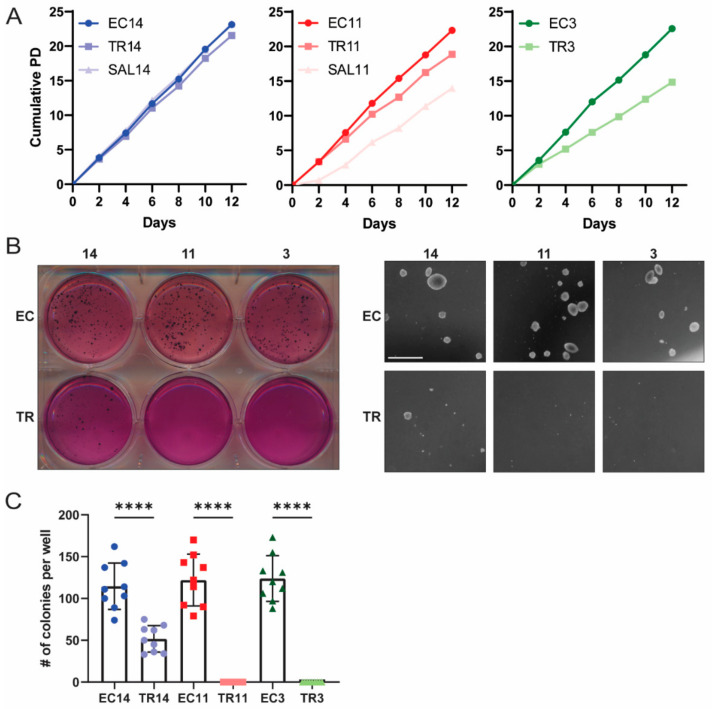
Thioridazine-differentiated cells have a reduced proliferation rate and reduced capacity for anchorage-independent growth. (**A**) Population doubling plots for the indicated EC cell lines and their thioridazine- and salinomycin-differentiated derivatives. (**B**) Representative whole-plate and microscopic images from soft agar growth assays assessing anchorage-independent growth of EC and thioridazine-differentiated cells. Scale bar = 1 mm (**C**) Quantification of soft agar growth assay experiments. The number of colonies was counted per well across three independent experiments, each with three technical replicates. Data are mean values ± standard deviation. **** *p* < 0.0001 (Mann–Whitney test).

EC cells have been established to be the CSCs of malignant teratocarcinomas, as the transplantation of a single EC cell to a mouse can form a tumor indistinguishable from the primary tumor [46]. Additionally, we previously reported that in the gPAK mouse model, the OCT4-expressing EC cells have tumor propagating activity, whereas bulk teratoma cells do not [23]. We therefore expected the EC cells to lose their tumorigenic potential following induced differentiation. To test this, we first assessed the ability of the differentiated cells to proliferate in normal two-dimensional as well as anchorage-independent conditions. Using a standard population doubling assay, we found that TR3, TR11, and SAL11 cells, but not TR14 or SAL14 cells, had a decreased proliferation rate compared to their parental EC cells (Figure 3A). All of the EC cells formed colonies when suspended in soft agar, whereas TR3 and TR11 cells were completely unable to do so. TR14 cells formed some colonies, but significantly fewer than their parental EC cells (Figure 3B,C). These results indicate that thioridazine-mediated differentiation of EC cells can slow their proliferation as well as reduce or eliminate their ability to grow under anchorage-independent conditions, an important hallmark of cancer.

**Figure 4:**
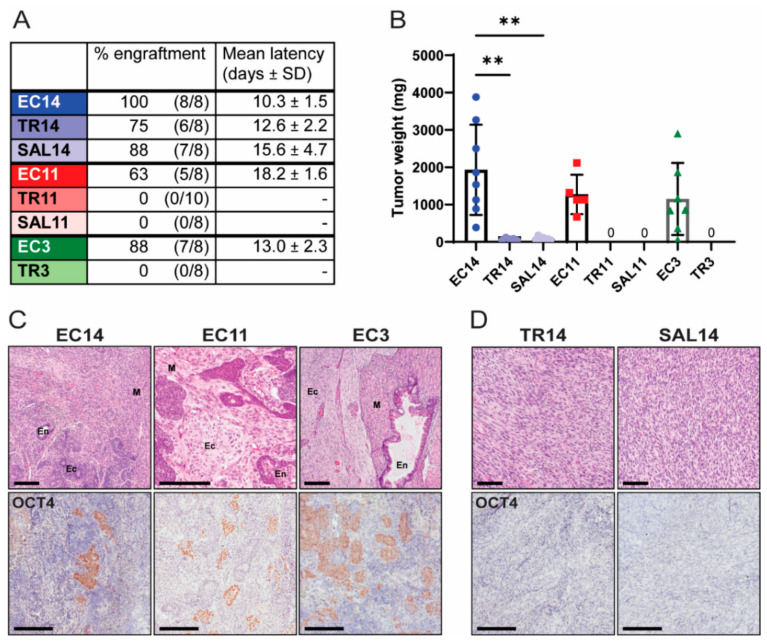
Thioridazine- and salinomycin-differentiated cells have reduced tumorigenic potential. (**A**) The indicated EC cells or the corresponding differentiated derivatives were injected subcutaneously into immune-compromised mice. Data show the percent engraftment (number of tumors formed/number of injection sites) and mean latency (time from cell injection until tumors were palpable). (**B**) Weight of tumors derived from EC or differentiated cells at endpoint (two weeks after initial tumor detection for EC14, TR14, SAL14, and EC3 cells, or three weeks after initial tumor detection for EC11 cells). TR11, SAL11, and TR3 cells did not form any tumors. Data are mean values ± standard deviation. ** *p* < 0.01 (Kruskal–Wallis test). (**C**) Representative images of H&E- and OCT4 IHC-stained tumors derived from EC cells. En = endoderm, M = mesoderm, Ec = ectoderm. Scale bars = 200 μm. (**D**) Representative images of H&E- and OCT4 IHC-stained tumors derived from TR14 and SAL14 cells. Scale bars = 200 μm.

To assess the tumorigenic potential of EC cells and their differentiated derivatives in vivo, we injected equal numbers of cells subcutaneously into immune-compromised mice. The mice were palpated regularly in order to accurately assess tumor latency. All EC cells, as well as TR14 and SAL14 cells, formed tumors in vivo, whereas TR11, SAL11, and TR3 cells did not (Figure 4A). The mice were euthanized two (EC3, EC14, TR14, and SAL14) or three (EC11) weeks after initial tumor detection, or 60 days after injection if tumors never formed (TR3, TR11, SAL11). TR14 and SAL14 cells formed tumors with an extended latency period, and at collection these tumors were significantly smaller than those formed by the parental EC14 cells. Specifically, the TR14 and SAL14 tumors were, on average, only 3.7% and 4.3% of the weight of EC14 tumors, respectively (Figure 4A,B). The tumors formed by EC cells were determined histologically to be malignant teratocarcinomas, as they contained teratoma tissue derived from all three embryonic germ layers (endoderm, mesoderm, and ectoderm) as well as OCT4-expressing embryonal carcinoma cells (Figure 4C). TR14 and SAL14 cells formed sarcomas that did not contain any OCT4-expressing cells (Figure 4D). Taken together, these data demonstrate that thioridazine- or salinomycin-mediated differentiation completely eliminated the tumorigenic potential of the EC cells in two out of three cell lines. TR14 and SAL14 cells did retain some anchorage independence and tumorgenicity, but the sarcomas formed by these cells lacked pluripotent EC cells and grew much slower than the tumors formed by EC14 cells. 

### 3.3. Thioridazine Treatment of Mice Bearing Transformed iPS Cell Allografts Reduces the Number of OCT4-Expressing Cells and Extends Survival

Finding that thioridazine and salinomycin induced differentiation of EC cells and greatly reduced their tumorigenic potential, we next sought to determine if thioridazine treatment of mice with malignant teratocarcinomas would eliminate EC cells in vivo and extend survival. We focused on thioridazine because it is an FDA-approved drug that could be repurposed for the treatment of malignant TGCTs, whereas salinomycin is not currently approved for use in humans. As EC cells are both malignantly transformed and pluripotent [46], we developed malignantly transformed induced pluripotent stem (iPS) cells as an additional experimental model of EC. Mouse embryonic fibroblasts (MEFs) with conditional *Pten* and *Kras* alleles were reprogrammed and then infected with a Cre-expressing adenovirus (Ad-Cre) to inactivate *Pten* and constitutively activate *Kras*. We anticipated that without targeting *Pten* and *Kras* the iPS cells would form benign teratomas, whereas the transformed cells would generate malignant teratocarcinomas. The tumorigenic potential of the normal (no Ad-Cre infection) and transformed (after Ad-Cre infection) iPS cells was assessed by injecting equal numbers of cells subcutaneously into opposite flanks of immune-compromised mice. At collection, the tumors formed by the transformed iPS cells were significantly larger than those formed by normal iPS cells (Supplementary Figure S3A). Both normal and transformed iPS cells formed tumors with teratoma components, but those formed by transformed iPS cells displayed evidence of malignancy such as necrosis and frequent mitotic figures (Supplementary Figure S3B). Tumors formed by both normal and transformed iPS cells contained OCT4-expressing cells, although those formed by transformed iPS cells contained significantly more (Supplementary Figure S3C,D).

**Figure 5:**
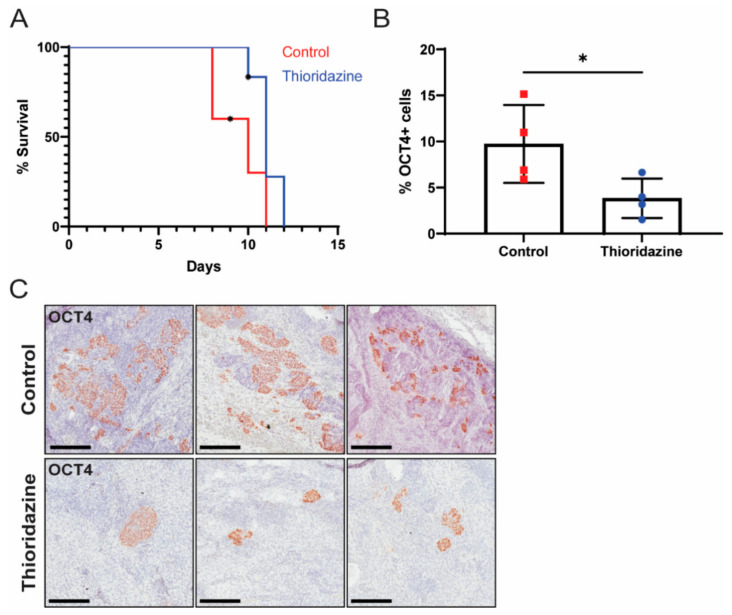
Thioridazine treatment of mice bearing transformed iPS cell allografts reduces the number of OCT4-expressing EC cells and extends survival. Nude mice were bilaterally injected with 1 million transformed iPS cells. When tumors were palpable, mice were treated via intraperitoneal injection with thioridazine (25 mg/kg) or vehicle alone (0.9% NaCl) every 3 days until tumors reached 2 cm in diameter or other humane endpoint criteria were met. (**A**) Kaplan–Meier survival curve depicting extension of survival in thioridazine-treated mice (*n* = 6) compared to control mice (*n* = 5). One control mouse, which was found dead, and two thioridazine-treated mice, which were euthanized for unrelated reasons, were censored (as indicated by an asterisk). Day zero is the day of first treatment. *p* = 0.0596 (Log-rank test). (**B**) Quantification of OCT4-positive cells in tumors from thioridazine-treated and control mice as determined by OCT4 IHC. Data are mean values ± standard deviation. * *p* < 0.05 (Unpaired *t*-test). (**C**) Representative images of OCT4 IHC-stained tumors from thioridazine-treated and control mice. Scale bars = 200 μm.

As the transformed iPS cells formed malignant teratocarcinomas with many OCT4-expressing cells in vivo, we next tested the ability of thioridazine to eliminate OCT4-expressing cells from tumors formed by transformed iPS cells and to extend the survival of mice bearing these tumors. Toward this end, nude mice were injected subcutaneously with transformed iPS cells. Once tumors were palpable, the mice were treated via intraperitoneal injection with thioridazine (25 mg/kg) or vehicle alone (0.9% NaCl) every three days until endpoint criteria were met. This dose and schedule of thioridazine administration, which was based on a prior report [47], moderately extended the survival of mice bearing transformed iPS cell allografts (Figure 5A). The survival extension with thioridazine treatment was not statistically significant (*p* = 0.0596), likely because these tumors grew very rapidly. Importantly, the tumors from the thioridazine-treated mice contained significantly fewer OCT4-expressing cells compared to the tumors from the control mice (Figure 5B,C). This suggests that thioridazine extends survival by targeting the highly malignant OCT4-expressing cells in transformed iPS cell allografts.

### 3.4. Thioridazine Treatment of Mice Bearing Human EC Cell Xenografts Extends Survival

**Figure 6:**
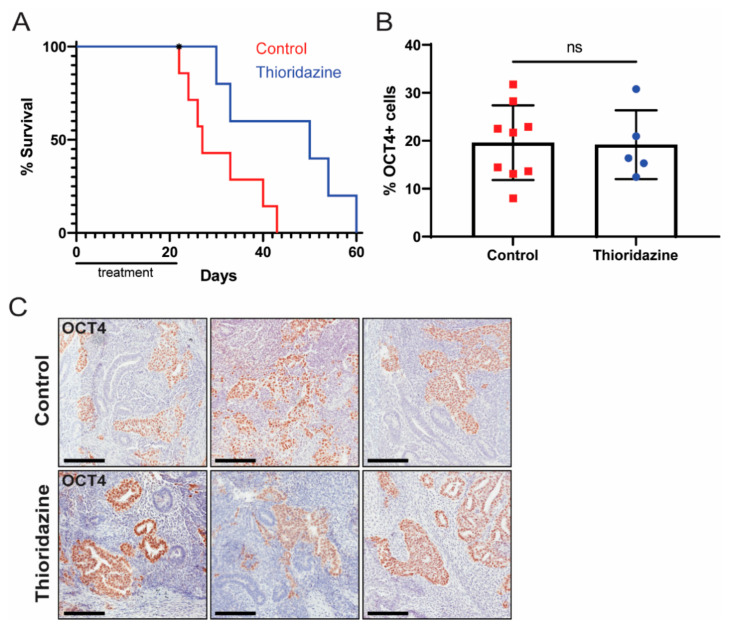
Thioridazine treatment of mice bearing human EC cell xenografts extends survival. Rag2KO mice were bilaterally injected with 10 million NT2D1 cells. When the tumors were palpable, mice were treated via intraperitoneal injection with thioridazine (25 mg/kg) or vehicle alone (0.9% NaCl) every three days for three weeks (seven doses total). Mice were euthanized when tumors reached 2 cm in diameter or other humane endpoint criteria were met. (**A**) Kaplan–Meier survival curve depicting extension of survival in thioridazine-treated mice (*n* = 6) compared to control mice (*n* = 7). One thioridazine-treated mouse was found dead and therefore censored (as indicated by an asterisk). *p* < 0.05 (Log-rank test). (**B**) Quantification of OCT4-positive cells in tumors from thioridazine-treated and control mice as determined by OCT4 IHC. Data are mean values ± standard deviation. ns = not significant (Unpaired *t*-test). (**C**) Representative images of OCT4 IHC-stained tumors from thioridazine-treated and control mice. Scale bars = 200 μm.

We next sought to test the therapeutic effects of thioridazine in a human EC xenograft model. Cells from the human EC cell line NT2D1, which is known to form malignant teratocarcinomas in vivo [48], were injected subcutaneously into Rag2KO mice. Once the tumors were palpable, mice were treated via intraperitoneal injection with thioridazine (25 mg/kg) or vehicle alone (0.9% NaCl) every three days for three weeks. Mice were euthanized when tumors reached 2 cm in diameter or other humane endpoint criteria were met. Thioridazine treatment significantly extended the survival of mice with NT2D1 xenografts (median survival of 50 days for thioridazine-treated mice, versus 27 days for control mice) (Figure 6A). There was no difference, however, in the percentage of OCT4-expressing cells in tumors from control versus thioridazine-treated mice (Figure 6B,C).

These data suggested that thioridazine may be an effective treatment against mixed germ cell tumors that contain EC cells. A primary motivation for identifying alternative therapies for TGCTs relates to the toxicity of the current standard-of-care regimens based on genotoxic chemotherapeutics [17]. Consistent with its activity as a dopamine receptor antagonist [49], many tumor-bearing mice that received thioridazine were sedated within an hour of administration, with some mice remaining sedated up to 24 h post-administration. However, mice that experienced the sedative effects of thioridazine recovered to normal function. Both nude and Rag2KO mice, which received transformed iPS and NT2D1 cells, respectively, that were treated with thioridazine, experienced some mild weight loss over the treatment period compared to control mice (Supplementary Figure S4A,B). This mild weight loss could be attributable to reduced feeding activity of thioridazine-treated mice due to the general sedation effects of the drug. When compared to mice treated with cisplatin, which commonly experience severe weight loss and a general decline in health, the side effects seen with thioridazine treatment were mild. Additionally, because temporary or permanent infertility remains a concern for patients receiving chemotherapy for TGCT treatment [50], we evaluated the effect of thioridazine treatment on the fertility of mice. As human patients are often diagnosed and treated with chemotherapy in their pre-reproductive to reproductive years, we randomly assigned wild-type male mice to thioridazine-treated or control groups and began treatment at weaning. Mice received thioridazine (25 mg/kg) or vehicle alone (0.9% NaCl) via intraperitoneal injection every three days for three weeks. At 7 weeks of age, each male was mated to two wild-type females for 12 weeks. There was no significant difference in average litter size between thioridazine-treated and control sires (Supplementary Figure S4C). These data suggest that treating mice with thioridazine prior to sexual maturity has no effect on fertility.

## 4. Discussion

TGCTs are highly curable with conventional genotoxic chemotherapy, but this treatment is associated with long-term morbidities that many TGCT patients, who are often treated as young men, face later in life [17]. Using mouse models of malignant nonseminomas, we investigated the ability of differentiation-inducing agents to target the tumor-propagating, pluripotent EC cells within these tumors, in order to slow tumor growth. Here, we have shown that thioridazine and salinomycin treatment differentiates cultured EC cells in vitro, as seen by a change in morphology and loss of pluripotency marker expression, and that this differentiation greatly reduces or eliminates the tumorigenic potential of these cells. These findings are consistent with the current understanding that EC cells are the CSCs of malignant teratocarcinomas, and that pure teratomas or teratoma components, which are the result of in vivo EC differentiation, lack tumor propagating ability [23,46].

The differentiated derivatives of one EC cell line, EC14, did retain some anchorage independence and tumorigenic potential, which is likely due to genetic differences between the EC cell lines rather than an incomplete loss of pluripotency, as the tumors formed by these cells were homogenous and did not contain any OCT4-positive pluripotent cells. While all three murine EC lines were *Pten*-null, only EC14 cells additionally expressed activated *Kras*^G12D^, although we cannot rule out the possibility that additional genetic or epigenetic alterations arose during tumor development or in vitro cultivation and also contributed to the differences observed between the independent EC cultures. Nevertheless, the results suggest that in some genetic contexts, the differentiation of the EC cells is sufficient to inhibit proliferation and result in a complete loss of tumorigenic potential, whereas in others the malignant phenotypes may be independent of pluripotency status and maintained after differentiation, at least to some extent. Similarly, TR14 and SAL14 cells, which retained some tumorigenic potential, may be good models for teratoma with malignant transformation, a rare phenomenon in which a teratomatous element of a nonseminoma transforms into a somatic malignancy such as a rhabdomyosarcoma or an adenocarcinoma [51]. Additional studies should be done to determine if and in what genetic context differentiation therapy for the treatment of TGCTs can result in malignant transformation of teratoma, as this would be a drawback to this treatment strategy.

As EC cells both have tumor-propagating activity and are amenable to differentiation, we hypothesized that differentiation therapy would be effective in treating mixed nonseminomas. Despite the very early findings in 1964 that EC cells are tumor-propagating [46], and in 1978 that they can be induced to differentiate by retinoic acid [52], differentiation therapy for the treatment of nonseminomas containing EC cells has not been explored in earnest to our knowledge. Differentiation therapy has been successfully implemented as early as 1990, before the modern concept of CSCs was well understood, when all-trans retinoic acid was reported to be effective in the treatment of acute promyelocytic leukemia [53]. Since then, CSCs have been identified in many hematologic and solid malignancies such as AML [54], breast [55], colon [56], and ovarian cancers [57]. Shortly after being identified, CSCs from many somatic tumor types were found to be intrinsically resistant to radiation and chemotherapy, often contributing to patient relapse [58,59,60]. In response, high-throughput drug screens were performed to identify compounds that target CSCs, such as those which identified thioridazine as selectively targeting neoplastic hPSCs [27], and salinomycin as selectively targeting breast CSCs [26].

The experiments presented here demonstrate that thioridazine can reduce the number of OCT4-expressing cells within malignant teratocarcinomas and extend the survival of tumor-bearing mice, including a human EC xenograft model, and may therefore be a good alternative or adjunctive treatment for malignant TGCTs. Although thioridazine-treated mice with NT2D1 xenografts survived significantly longer than control mice, there was no difference in the percentage of OCT4-expressing cells within the tumors of the two groups. This was likely due to the fact that these mice survived well beyond the end of the treatment period, and any OCT4-expressing cells remaining after treatment would have had time to repopulate the tumor, especially given that thioridazine-treated mice lived longer with tumors than control mice, on average. We do recognize that the current standard of care regimen, featuring cisplatin-based chemotherapy, very often cures patients of TGCTs. Interestingly, the effects of cisplatin and thioridazine on malignant teratocarcinomas in mice are similar in that both target OCT4-expressing EC cells within a tumor [23]. We propose that using thioridazine in combination with low-dose chemotherapy may achieve similar therapeutic efficacy while mitigating the short- and long-term side effects of high-dose chemotherapy.

Additionally, thioridazine alone or in combination with other therapeutics may be effective in treating chemoresistant TGCTs. Chemoresistance in TCGTs is associated with a loss of pluripotency markers OCT4 and NANOG; however, it is unclear whether the loss of these markers is a driver of chemoresistance or is secondary to an independent process underlying the acquisition of chemoresistance [61,62]. Interestingly, EC cells showing typical morphology but lacking OCT4 have been identified in xenografts formed by a cisplatin-resistant nonseminoma cell line [63]. This suggests that OCT4 loss may not completely abrogate the stem cell phenotype of EC cells and therefore, chemoresistant EC cells and EC-containing tumors may still be amenable to differentiation. Additionally, thioridazine, in combination with chemotherapeutics, may have antineoplastic effects against tumors which have developed chemoresistance, as it has been found to sensitize chemoresistant lung and ovarian cancer cells to cisplatin [64], glioblastoma cells to temozolomide [65], and head and neck cancer cells to carboplatin [66].

Thioridazine, which produces antipsychotic effects mainly through antagonism of the D2 dopamine receptor (DRD2) [49], has been found to stimulate diverse antineoplastic effects in a range of malignant cells through a variety of mechanisms, both DRD2-dependent and -independent. Neoplastic hPSCs induced to differentiate by thioridazine express all five dopamine receptors, whereas normal hPSCs, which thioridazine has no effect on, were devoid of dopamine receptor expression [27]. In breast cancer cells, thioridazine has been found to inhibit self-renewal via DRD2-dependent STAT3 inhibition as well as induce a G1 cell cycle arrest independent of DRD2 [67]. Thioridazine can also induce apoptosis as well as both promote and inhibit autophagy, depending on the cancer context, although it is unclear whether these effects are mediated by DRD2 antagonism [65,68,69]. In a phase 1 clinical trial evaluating the use of thioridazine in combination with cytarabine in AML patients, the extent of leukemic blast reduction after thioridazine treatment was associated with expression of DRD2 on leukemic cells [70], suggesting that thioridazine’s ability to inhibit CSC function is downstream of DRD2 antagonism. The assessment of dopamine receptor expression in EC cells and further elucidation of the mechanism of action of thioridazine in this context represent areas for future experimentation.

The mechanism by which thioridazine induces the differentiation of CSCs is largely unknown; however, a recent study demonstrated that thioridazine elevates cAMP levels in AML progenitor cells through DRD2 engagement, which induces cell maturation [71]. Additionally, thioridazine induces the differentiation of prostate CSCs by inhibiting the phosphorylation of AMPK [72]. A better understanding of the mechanism through which thioridazine induces EC cell differentiation may allow for a more targeted approach. Additionally, thioridazine and other DRD2 antagonists could be modified to improve tolerability, as dose-limiting toxicities such as cardiac arrhythmias and neurologic events have been reported in patients given thioridazine for AML treatment [70]. In light of the data presented here, we believe that further development of differentiation therapy approaches for the treatment of mixed nonseminomas holds promise for reducing the long-term adverse effects of cisplatin-based chemotherapy commonly faced by TGCT survivors.

## Data Availability

The RNA-sequencing data presented in this studyhas been deposited in the Gene Expression Omnibus (GSE168261).

## Supplementary Materials

The following are available online at https://www.mdpi.com/article/10.3390/cancers13092045/s1, Figure S1: Differential expression analysis of murine EC cells and their thioridazine-differentiated derivatives; Figure S2: Gene ontology analysis of differentially expressed genes between murine EC cells and their thioridazine-differentiated derivatives; Figure S3: Pten inactivation and Kras activation causes malignant transformation in induced pluripotent stem cells; Figure S4: Thioridazine treatment causes mild weight loss but does not compromise fertility in mice; Figure S5: Original Western blot images.

## References

[1] Winter C., Albers P. (2011). Testicular germ cell tumors: Pathogenesis, diagnosis and treatment. Nat. Rev. Endocrinol..

[2] Cheng L., Albers P., Berney D.M., Feldman D.R., Daugaard G., Gilligan T., Looijenga L.H.J. (2018). Testicular cancer. Nat. Rev. Dis. Primers.

[3] Schmidtova S., Kalavska K., Kucerova L. (2018). Molecular Mechanisms of Cisplatin Chemoresistance and Its Circumventing in Testicular Germ Cell Tumors. Curr. Oncol. Rep..

[4] Epstein J.I., Lotan T.L., Kumar V., Abbas A.K., Aster J.C. (2015). The Lower Urinary Tract and Male Genital System. Robbins and Cotran Pathologic Basis of Disease.

[5] Ulbright T.M. (2005). Germ cell tumors of the gonads: A selective review emphasizing problems in differential diagnosis, newly appreciated, and controversial issues. Mod. Pathol..

[6] Daugaard G., Gundgaard M.G., Mortensen M.S., Agerbæk M., Holm N.V., Rørth M., Von Der Maase H., Christensen I.J., Lauritsen J. (2014). Surveillance for Stage I Nonseminoma Testicular Cancer: Outcomes and Long-Term Follow-Up in a Population-Based Cohort. J. Clin. Oncol..

[7] Kier M.G., Lauritsen J., Mortensen M.S., Bandak M., Andersen K.K., Hansen M.K., Agerbaek M., Holm N.V., Dalton S.O., Johansen C. (2017). Prognostic Factors and Treatment Results After Bleomycin, Etoposide, and Cisplatin in Germ Cell Cancer: A Population-based Study. Eur. Urol..

[8] Mortensen M.S., Lauritsen J., Gundgaard M.G., Agerbæk M., Holm N.V., Christensen I.J., von der Maase H., Daugaard G. (2014). A Nationwide Cohort Study of Stage I Seminoma Patients Followed on a Surveillance Program. Eur. Urol..

[9] Bloom J.C., Loehr A.R., Schimenti J.C., Weiss R.S. (2019). Germline genome protection: Implications for gamete quality and germ cell tumorigenesis. Andrology.

[10] Singh R., Fazal Z., Freemantle S.J., Spinella M.J. (2019). Mechanisms of cisplatin sensitivity and resistance in testicular germ cell tumors. Cancer Drug Resist..

[11] Meyts E.R.-D., McGlynn K.A., Okamoto K., Jewett M.A.S., Bokemeyer C. (2016). Testicular germ cell tumours. Lancet.

[12] Nurgali K., Jagoe R.T., Abalo R. (2018). Editorial: Adverse Effects of Cancer Chemotherapy: Anything New to Improve Tolerance and Reduce Sequelae?. Front. Pharmacol..

[13] Dearnaley D.P., Huddart R.A., Horwich A. (2001). Managing testicular cancer. BMJ.

[14] Travis L.B., Beard C., Allan J.M., Dahl A.A., Feldman D.R., Oldenburg J., Daugaard G., Kelly J.L., Dolan M.E., Hannigan R. (2010). Testicular cancer survivorship: Research strategies and recommendations. J. Natl. Cancer Inst..

[15] Fung C., Fossa S.D., Williams A., Travis L.B. (2015). Long-term Morbidity of Testicular Cancer Treatment. Urol. Clin. N. Am..

[16] Schepisi G., De Padova S., De Lisi D., Casadei C., Meggiolaro E., Ruffilli F., Rosti G., Lolli C., Ravaglia G., Conteduca V. (2019). Psychosocial Issues in Long-Term Survivors of Testicular Cancer. Front. Endocrinol..

[17] Agrawal V., Dinh P.C., Fung C., Monahan P.O., Althouse S.K., Norton K., Cary C., Einhorn L., Fossa S.D., Adra N. (2020). Adverse Health Outcomes among US Testicular Cancer Survivors after Cisplatin-Based Chemotherapy vs Surgical Management. JNCI Cancer Spectr..

[18] Reya T., Morrison S.J., Clarke M.F., Weissman I.L. (2001). Stem cells, cancer, and cancer stem cells. Nature.

[19] Sell S. (2004). Stem cell origin of cancer and differentiation therapy. Crit. Rev. Oncol./Hematol..

[20] Lambert A.W., Pattabiraman D.R., Weinberg R.A. (2017). Emerging Biological Principles of Metastasis. Cell.

[21] Hanahan D., Weinberg R.A. (2011). Hallmarks of Cancer: The Next Generation. Cell.

[22] Sesterhenn I.A., Davis J.C. (2004). Pathology of Germ Cell Tumors of the Testis. Cancer Control.

[23] Pierpont T.M., Lyndaker A.M., Anderson C.M., Jin Q., Moore E.S., Roden J.L., Braxton A., Bagepalli L., Kataria N., Hu H.Z. (2017). Chemotherapy-Induced Depletion of OCT4-Positive Cancer Stem Cells in a Mouse Model of Malignant Testicular Cancer. Cell Rep..

[24] Batool A., Karimi N., Wu X.-N., Chen S.-R., Liu Y.-X. (2019). Testicular germ cell tumor: A comprehensive review. Cell. Mol. Life Sci..

[25] Kevin D.A., Meujo D.A., Hamann M.T. (2009). Polyether ionophores: Broad-spectrum and promising biologically active molecules for the control of drug-resistant bacteria and parasites. Expert Opin. Drug Discov..

[26] Gupta P.B., Onder T.T., Jiang G., Tao K., Kuperwasser C., Weinberg R.A., Lander E.S. (2009). Identification of selective inhibitors of cancer stem cells by high-throughput screening. Cell.

[27] Sachlos E., Risueño R.M., Laronde S., Shapovalova Z., Lee J.-H., Russell J., Malig M., McNicol J.D., Fiebig-Comyn A., Graham M. (2012). Identification of Drugs Including a Dopamine Receptor Antagonist that Selectively Target Cancer Stem Cells. Cell.

[28] Driver J.A., Logroscino G., Buring J.E., Gaziano J.M., Kurth T. (2007). A Prospective Cohort Study of Cancer Incidence Following the Diagnosis of Parkinson’s Disease. Cancer Epidemiol. Biomark. Prev..

[29] Dalton S.O., Mellemkjær L., Thomassen L., Mortensen P.B., Johansen C. (2005). Risk for cancer in a cohort of patients hospitalized for schizophrenia in Denmark, 1969–1993. Schizophr. Res..

[30] Lyndaker A.M., Pierpont T.M., Loehr A.R., Weiss R.S. (2021). A Genetically Engineered Mouse Model of Malignant Testicular Germ Cell Tumors. Methods Mol. Biol..

[31] National Research Council (2011). Guide for the Care and Use of Laboratory Animals: Eighth Edition.

[32] Todaro G.J., Green H. (1963). Quantitative studies of the growth of mouse embryo cells in culture and their development into established lines. J. Cell Biol..

[33] Schnabel L.V., Abratte C.M., Schimenti J.C., Southard T.L., Fortier L.A. (2012). Genetic background affects induced pluripotent stem cell generation. Stem. Cell Res. Ther..

[34] Zhu M., Weiss R.S. (2007). Increased common fragile site expression, cell proliferation defects, and apoptosis following conditional inactivation of mouse Hus1 in primary cultured cells. Mol. Biol. Cell.

[35] Martin M. (2011). Cutadapt removes adapter sequences from high-throughput sequencing reads. EMBnet J..

[36] Dobin A., Davis C.A., Schlesinger F., Drenkow J., Zaleski C., Jha S., Batut P., Chaisson M., Gingeras T.R. (2013). STAR: Ultrafast universal RNA-seq aligner. Bioinformatics.

[37] Varet H., Brillet-Gueguen L., Coppee J.Y., Dillies M.A. (2016). SARTools: A DESeq2- and EdgeR-Based R Pipeline for Comprehensive Differential Analysis of RNA-Seq Data. PLoS ONE.

[38] Love M.I., Huber W., Anders S. (2014). Moderated estimation of fold change and dispersion for RNA-seq data with DESeq2. Genome Biol..

[39] Ge S.X., Son E.W., Yao R. (2018). iDEP: An integrated web application for differential expression and pathway analysis of RNA-Seq data. BMC Bioinform..

[40] Cerami E., Gao J., Dogrusoz U., Gross B.E., Sumer S.O., Aksoy B.A., Jacobsen A., Byrne C.J., Heuer M.L., Larsson E. (2012). The cBio cancer genomics portal: An open platform for exploring multidimensional cancer genomics data. Cancer Discov..

[41] Gao J., Aksoy B.A., Dogrusoz U., Dresdner G., Gross B., Sumer S.O., Sun Y., Jacobsen A., Sinha R., Larsson E. (2013). Integrative analysis of complex cancer genomics and clinical profiles using the cBioPortal. Sci. Signal..

[42] Subramanian A., Tamayo P., Mootha V.K., Mukherjee S., Ebert B.L., Gillette M.A., Paulovich A., Pomeroy S.L., Golub T.R., Lander E.S. (2005). Gene set enrichment analysis: A knowledge-based approach for interpreting genome-wide expression profiles. Proc. Natl. Acad. Sci. USA.

[43] Mi H., Ebert D., Muruganujan A., Mills C., Albou L.P., Mushayamaha T., Thomas P.D. (2021). PANTHER version 16: A revised family classification, tree-based classification tool, enhancer regions and extensive API. Nucleic Acids Res..

[44] Andrews P.W., Gonczol E., Plotkin S.A., Dignazio M., Oosterhuis J.W. (1986). Differentiation of TERA-2 human embryonal carcinoma cells into neurons and HCMV permissive cells: Induction by agents other than retinoic acid. Differentiation.

[45] Mehravar M., Ghaemimanesh F., Poursani E.M. (2021). An Overview on the Complexity of OCT4: At the Level of DNA, RNA and Protein. Stem Cell Rev. Rep..

[46] Kleinsmith L.J., Pierce G.B. (1964). Multipotentiality of Single Embryonal Carcinoma Cells. Cancer Res..

[47] Yong M., Yu T., Tian S., Liu S., Xu J., Hu J., Hu L. (2017). DR2 blocker thioridazine: A promising drug for ovarian cancer therapy. Oncol. Lett..

[48] Andrews P.W., Damjanov I., Simon D., Banting G.S., Carlin C., Dracopoli N.C., Fogh J. (1984). Pluripotent embryonal carcinoma clones derived from the human teratocarcinoma cell line Tera-2. Differentiation in vivo and in vitro. Lab. Investig..

[49] Varga B., Csonka Á., Csonka A., MolnÁR J., Amaral L., Spengler G. (2017). Possible Biological and Clinical Applications of Phenothiazines. Anticancer Res..

[50] Dohle G.R. (2010). Male infertility in cancer patients: Review of the literature. Int. J. Urol..

[51] Cabral F.C., Krajewski K.M., Rosenthal M.H., Hirsch M.S., Howard S.A. (2014). Teratoma with malignant transformation: Report of three cases and review of the literature. Clin. Imaging.

[52] Strickland S., Mahdavi V. (1978). The induction of differentiation in teratocarcinoma stem cells by retinoic acid. Cell.

[53] Castaigne S., Chomienne C., Daniel M.T., Ballerini P., Berger R., Fenaux P., Degos L. (1990). All-trans retinoic acid as a differentiation therapy for acute promyelocytic leukemia. I. Clinical results. Blood.

[54] Bonnet D., Dick J.E. (1997). Human acute myeloid leukemia is organized as a hierarchy that originates from a primitive hematopoietic cell. Nat. Med..

[55] Al-Hajj M., Wicha M.S., Benito-Hernandez A., Morrison S.J., Clarke M.F. (2003). Prospective identification of tumorigenic breast cancer cells. Proc. Natl. Acad. Sci. USA.

[56] O’Brien C.A., Pollett A., Gallinger S., Dick J.E. (2007). A human colon cancer cell capable of initiating tumour growth in immunodeficient mice. Nature.

[57] Zhang S., Balch C., Chan M.W., Lai H.C., Matei D., Schilder J.M., Yan P.S., Huang T.H., Nephew K.P. (2008). Identification and characterization of ovarian cancer-initiating cells from primary human tumors. Cancer Res..

[58] Li X., Lewis M.T., Huang J., Gutierrez C., Osborne C.K., Wu M.F., Hilsenbeck S.G., Pavlick A., Zhang X., Chamness G.C. (2008). Intrinsic resistance of tumorigenic breast cancer cells to chemotherapy. J. Natl. Cancer Inst..

[59] Bao S., Wu Q., McLendon R.E., Hao Y., Shi Q., Hjelmeland A.B., Dewhirst M.W., Bigner D.D., Rich J.N. (2006). Glioma stem cells promote radioresistance by preferential activation of the DNA damage response. Nature.

[60] Zhao J. (2016). Cancer stem cells and chemoresistance: The smartest survives the raid. Pharmacol. Ther..

[61] Taylor-Weiner A., Zack T., O’Donnell E., Guerriero J.L., Bernard B., Reddy A., Han G.C., AlDubayan S., Amin-Mansour A., Schumacher S.E. (2016). Genomic evolution and chemoresistance in germ-cell tumours. Nature.

[62] Mueller T., Mueller L.P., Luetzkendorf J., Voigt W., Simon H., Schmoll H.J. (2006). Loss of Oct-3/4 expression in embryonal carcinoma cells is associated with induction of cisplatin resistance. Tumor Biol..

[63] Mueller T., Mueller L.P., Holzhausen H.J., Witthuhn R., Albers P., Schmoll H.J. (2010). Histological evidence for the existence of germ cell tumor cells showing embryonal carcinoma morphology but lacking OCT4 expression and cisplatin sensitivity. Histochem. Cell Biol..

[64] Qian G., Dai L., Yu T. (2019). Thioridazine Sensitizes Cisplatin against Chemoresistant Human Lung and Ovary Cancer Cells. DNA Cell Biol..

[65] Johannessen T.C., Hasan-Olive M.M., Zhu H., Denisova O., Grudic A., Latif M.A., Saed H., Varughese J.K., Rosland G.V., Yang N. (2019). Thioridazine inhibits autophagy and sensitizes glioblastoma cells to temozolomide. Int. J. Cancer.

[66] Seo S.U., Cho H.K., Min K.J., Woo S.M., Kim S., Park J.W., Kim S.H., Choi Y.H., Keum Y.S., Hyun J.W. (2017). Thioridazine enhances sensitivity to carboplatin in human head and neck cancer cells through downregulation of c-FLIP and Mcl-1 expression. Cell Death Dis..

[67] Tegowski M., Fan C., Baldwin A.S. (2018). Thioridazine inhibits self-renewal in breast cancer cells via DRD2-dependent STAT3 inhibition, but induces a G1 arrest independent of DRD2. J. Biol. Chem..

[68] Chu C.W., Ko H.J., Chou C.H., Cheng T.S., Cheng H.W., Liang Y.H., Lai Y.L., Lin C.Y., Wang C., Loh J.K. (2019). Thioridazine Enhances P62-Mediated Autophagy and Apoptosis Through Wnt/β-Catenin Signaling Pathway in Glioma Cells. Int. J. Mol. Sci..

[69] Kang S., Dong S.M., Kim B.R., Park M.S., Trink B., Byun H.J., Rho S.B. (2012). Thioridazine induces apoptosis by targeting the PI3K/Akt/mTOR pathway in cervical and endometrial cancer cells. Apoptosis.

[70] Aslostovar L., Boyd A.L., Almakadi M., Collins T.J., Leong D.P., Tirona R.G., Kim R.B., Julian J.A., Xenocostas A., Leber B. (2018). A phase 1 trial evaluating thioridazine in combination with cytarabine in patients with acute myeloid leukemia. Blood Adv..

[71] Aslostovar L., Boyd A.L., Benoit Y.D., Di Lu J., Rodriguez J.L.G., Nakanishi M., Porras D.P., Reid J.C., Mitchell R.R., Leber B. (2021). Abnormal dopamine receptor signaling allows selective therapeutic targeting of neoplastic progenitors in AML patients. Cell Rep. Med..

[72] Lee S.I., Roney M.S., Park J.H., Baek J.Y., Park J., Kim S.K., Park S.K. (2019). Dopamine receptor antagonists induce differentiation of PC-3 human prostate cancer cell-derived cancer stem cell-like cells. Prostate.

