# Supplementary material for: Targeting Cancer Stem Cells with Differentiation Agents as an Alternative to Genotoxic Chemotherapy for the Treatment of Malignant Testicular Germ Cell Tumors
Source: Cancers (Basel). 2021 Apr 23;13(9):2045. doi: 10.3390/cancers13092045 (PMC8122873; doi:10.3390/cancers13092045)
Supplement: Supplementary file 1 [file cancers-13-02045-s001.zip › cancers-1130993-supplementary.pdf]

SUPPLEMENTARY DATA

Targeting cancer stem cells with differentiation agents as an alternative to genotoxic chemotherapy for the treatment of malignant testicular germ cell tumors

Amanda R. Loehr, Timothy M. Pierpont, Eric Gelsleichter, Anabella Maria D. Galang, Irma R. Fernandez, Elizabeth S. Moore, Matthew Z. Guo, Andrew D. Miller, and Robert S. Weiss

5 Supplementary figures

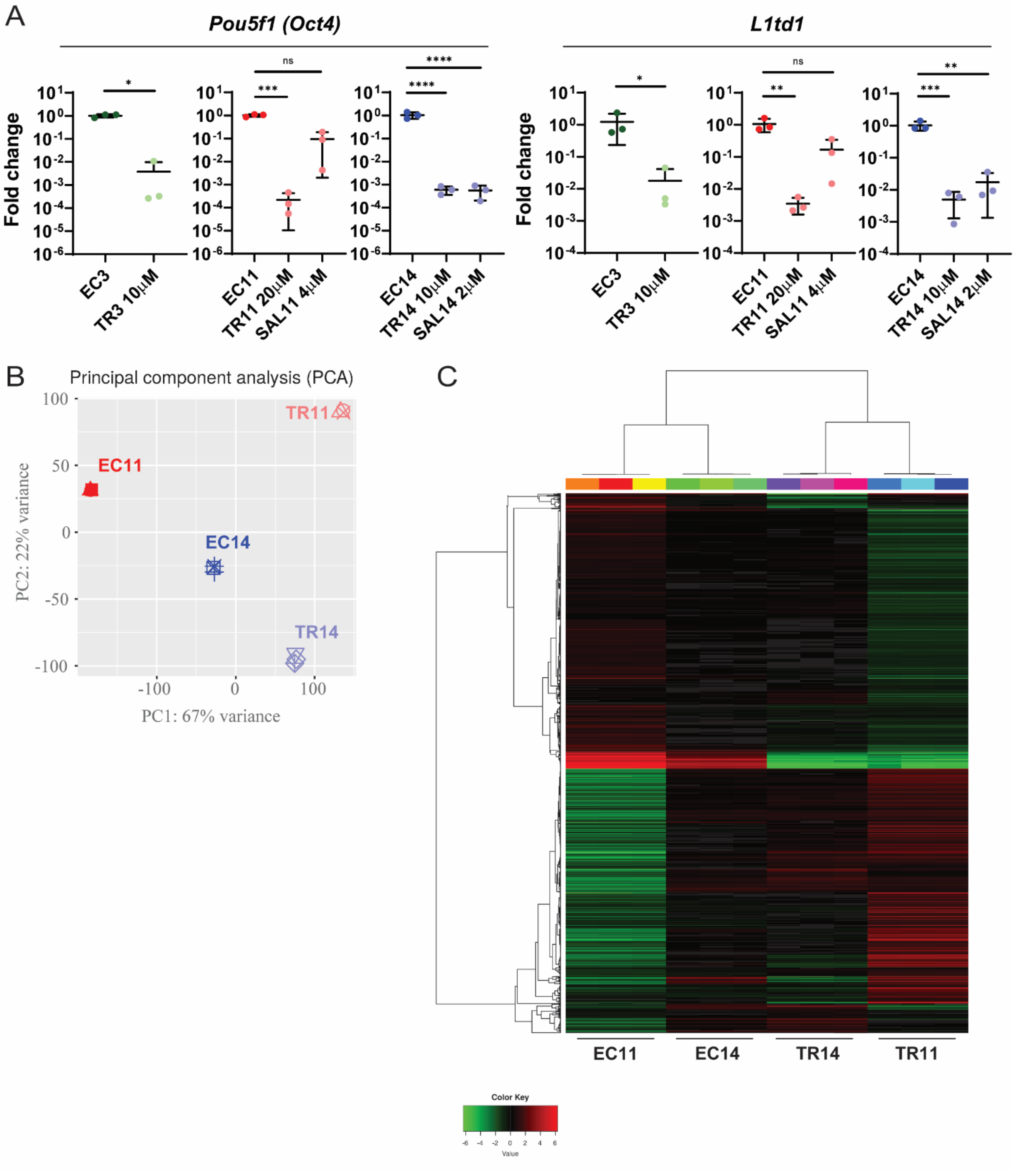

Supplementary Figure S1. Differential expression analysis of murine EC cells and their thioridazine-differentiated derivatives. (A) Quantitative PCR analysis of *Pouf51* (encodes OCT4) and *L1td1* in murine EC cells and their thioridazine- and salinomycin-differentiated derivatives. Data are mean values ± standard deviation. A lower error bar is not shown if the value

would extend to a negative number. Each data point represents an independent biological experiment (n= 3), and is the average of three technical replicates per experiment. When comparing two groups, a Welch's t-test was performed. When comparing three groups, a one-way ANOVA and Tukey's test for multiple comparisons was performed. ns = not significant, * $p < 0.05$, ** $p < 0.01$, *** $p < 0.001$, **** $p < 0.0001$. (B) Principal Component Analysis of RNASeq data for two murine EC cell lines and their thioridazine-differentiated derivatives. Three independent replicates were analyzed per group. (C). Hierarchical clustering heatmap for the top 500 most differentially expressed genes in two murine EC cell lines and their thioridazine-differentiated derivatives. Three independent replicates were analyzed per group.

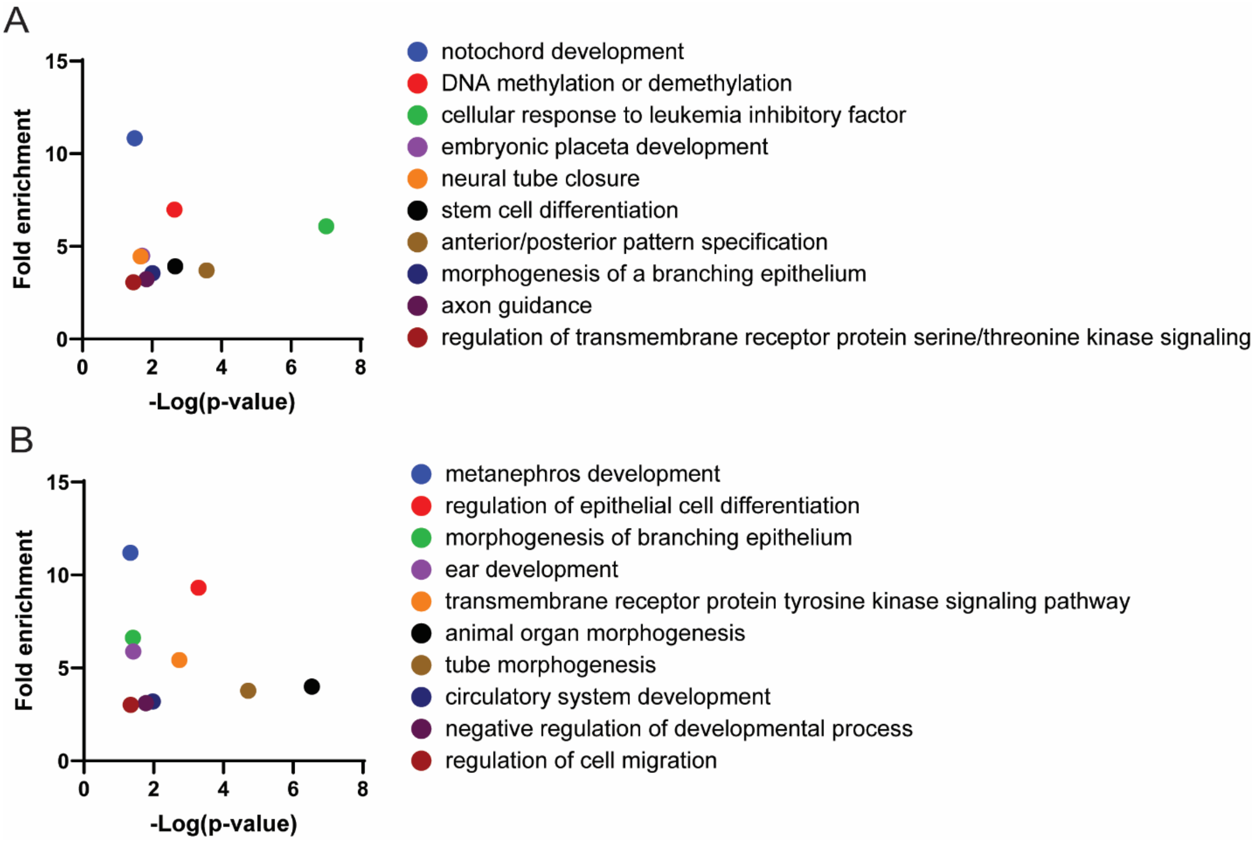

Supplementary Figure S2. Gene Ontology analysis of differentially expressed genes between murine EC cells and their thioridazine-differentiated derivatives. Gene lists containing genes that were significantly upregulated (FDR < 0.05, normalized count ≥ 50 in at least one group, and $\log_2$(fold change) ≥ 1) in both EC11 and EC14 compared to their differentiated derivates and those that were significantly upregulated in both TR11 and TR14 compared to their parental EC lines were used in a PANTHER Overrepresentation Test. Graphs show the top 10 distinct GO Biological Processes overrepresented in (A) EC cells and (B) differentiated derivatives. Fold enrichment describes the number of genes in our gene list that map to that GO term divided by the number of genes that would be expected to map to the GO term based on random whole-genome sampling. P-values were determined by Fisher's Exact test with Bonferroni correction for multiple testing.

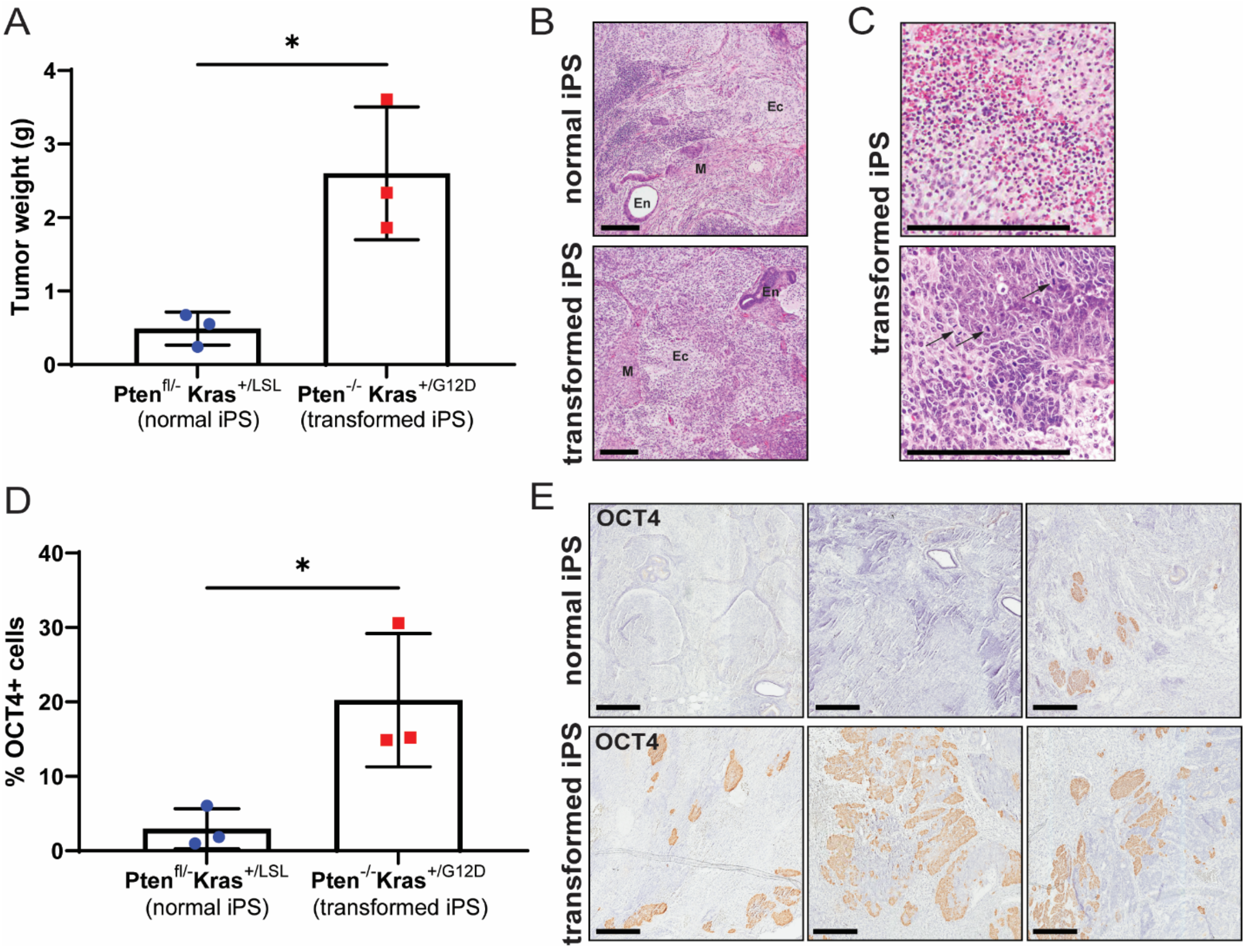

Supplementary Figure S3. *Pten* inactivation and *Kras* activation causes malignant transformation in induced pluripotent stem cells. Nude mice were injected with 1 million normal iPS cells on the left flank, and 1 million transformed iPS cells on the right flank, and collected when a tumor reached 2 cm in diameter. (A) Graphs show the weight of tumors derived from normal ($n = 3$) or transformed ($n = 3$) iPS cells at endpoint. Data are mean values ± standard deviation. * $p < 0.05$ (Unpaired t-test). (B) Representative images of H&E-stained tumors derived from normal or transformed iPS cells. En = endoderm, M = mesoderm, Ec = ectoderm. Scale bars = 200 μm. (C) Representative images of H&E-stained tumors derived from transformed iPS cells showing necrosis (top) and mitotic cells (bottom, arrows). Scale bars = 200 μm. (D) Quantification of OCT4-positive cells in tumors derived from normal or transformed iPS cells. Data are mean

values ± standard deviation. * $p < 0.05$ (Unpaired t-test). (E) Representative images of OCT4 IHC-stained tumors derived from normal or transformed iPS cells. Scale bars = 300 μm.

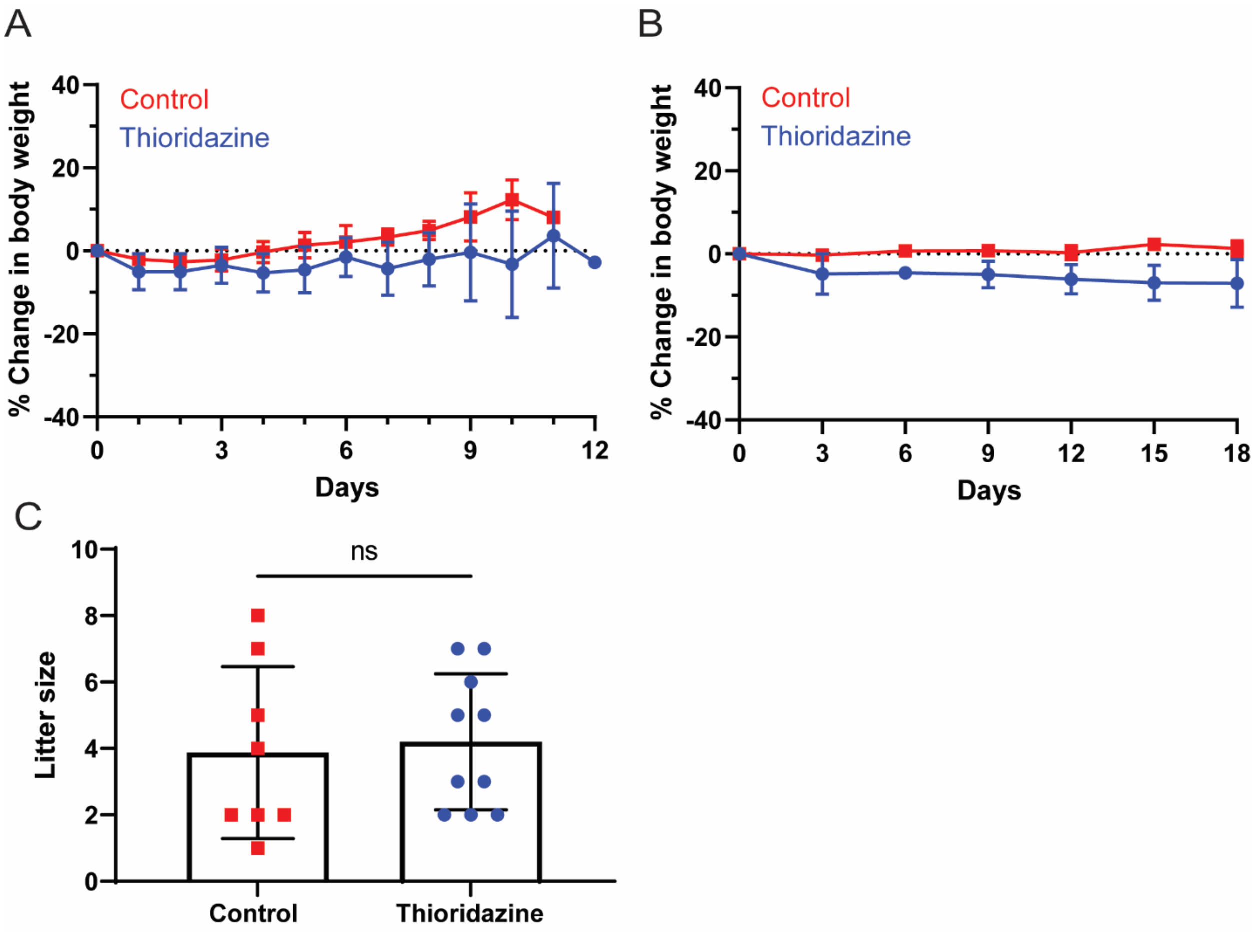

Supplementary Figure S4. Thioridazine treatment causes mild weight loss but does not compromise fertility in mice. (A) Percent change in body weight over time was plotted for thioridazine-treated (n = 6) or control (n = 5) mice bearing transformed iPS cell allografts. Data are mean values ± standard deviation. (B) Percent change in body weight over time was plotted for thioridazine-treated (n = 5) or control (n = 7) mice bearing NT2D1 xenografts. Data are mean values ± standard deviation. (C) For assessment of the effects of thioridazine treatment on fertility, wild-type mice were randomly assigned to thioridazine-treated or control groups and began treatment at weaning. Thioridazine-treated mice received 25 mg/kg thioridazine in 0.9% NaCl via intraperitoneal injection every 3 days for 3 weeks. Control mice received 0.9% NaCl in the same manner. At 7 weeks of age, each male was mated to two wild-type females for 12 weeks. Graph

shows the number of pups per litter from these matings. n = 2 males per group. Data are mean values ± standard deviation. ns = not significant (Unpaired t-test).

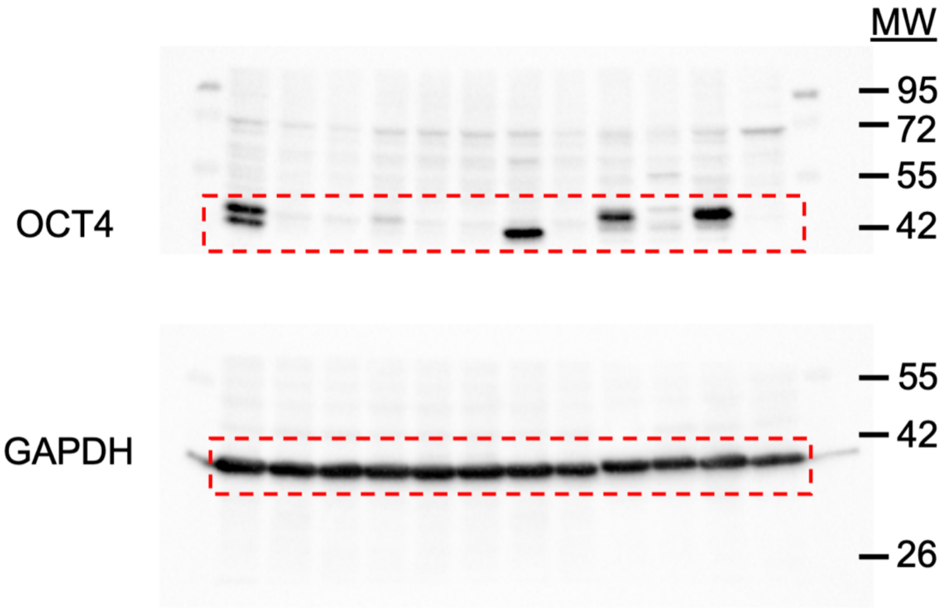

Supplementary Figure S5. Original western blot images. Red dotted boxes represent the areas that were cropped for Figure 1C.
